# 3D Printing of Drug-Loaded Thermoplastic Polyurethane Meshes: A Potential Material for Soft Tissue Reinforcement in Vaginal Surgery

**DOI:** 10.3390/pharmaceutics12010063

**Published:** 2020-01-13

**Authors:** Juan Domínguez-Robles, Caterina Mancinelli, Elena Mancuso, Inmaculada García-Romero, Brendan F. Gilmore, Luca Casettari, Eneko Larrañeta, Dimitrios A. Lamprou

**Affiliations:** 1 School of Pharmacy, Queen’s University Belfast, Lisburn Road 97, Belfast BT9 7BL, UK; j.dominguezrobles@qub.ac.uk (J.D.-R.); c.mancinelli3@campus.uniurb.it (C.M.); b.gilmore@qub.ac.uk (B.F.G.); 2 Department of Biomolecular Sciences, University of Urbino Carlo Bo, Piazza del Rinascimento 6, 61029 Urbino, Italy; luca.casettari@uniurb.it; 3 Nanotechnology and Integrated Bio-Engineering Centre (NIBEC), Ulster University, Jordanstown Campus, Jordanstown BT37 0QB, UK; e.mancuso@ulster.ac.uk; 4 Wellcome-Wolfson Institute for Experimental Medicine, Queen’s University Belfast, Belfast BT9 7BL, UK; I.Garcia-Romero@qub.ac.uk

**Keywords:** 3D printing, fused deposition modelling, extrusion, vaginal meshes, mechanical properties, drug release, anti-infective devices, pelvic organ prolapse, stress urinary incontinence

## Abstract

Current strategies to treat pelvic organ prolapse (POP) or stress urinary incontinence (SUI), include the surgical implantation of vaginal meshes. Recently, there have been multiple reports of issues generated by these meshes conventionally made of poly(propylene). This material is not the ideal candidate, due to its mechanical properties leading to complications such as chronic pain and infection. In the present manuscript, we propose the use of an alternative material, thermoplastic polyurethane (TPU), loaded with an antibiotic in combination with fused deposition modelling (FDM) to prepare safer vaginal meshes. For this purpose, TPU filaments containing levofloxacin (LFX) in various concentrations (e.g., 0.25%, 0.5%, and 1%) were produced by extrusion. These filaments were used to 3D print vaginal meshes. The printed meshes were fully characterized through different tests/analyses such as fracture force studies, attenuated total reflection-Fourier transform infrared, thermal analysis, scanning electron microscopy, X-ray microcomputed tomography (μCT), release studies and microbiology testing. The results showed that LFX was uniformly distributed within the TPU matrix, regardless the concentration loaded. The mechanical properties showed that poly(propylene) (PP) is a tougher material with a lower elasticity than TPU, which seemed to be a more suitable material due to its elasticity. In addition, the printed meshes showed a significant bacteriostatic activity on both *Staphylococcus aureus* and *Escherichia coli* cultures, minimising the risk of infection after implanting them. Therefore, the incorporation of LFX to the TPU matrix can be used to prepare anti-infective vaginal meshes with enhanced mechanical properties compared with current PP vaginal meshes.

## 1. Introduction

Pelvic organ prolapse (POP) and stress urinary incontinence (SUI), are two very common disorders affecting 30–40% of women worldwide, mainly with the increase in age [1]. Since the population is growing gradually older, with the passage of time there will be an increase in the incidence of POP of 46% between 2010 and 2050 [2]. Although they are not lethal diseases, these two pathologies negatively influence the quality of life of women, including their social, sexual, physical and psychological well-being [1,3]. The implantation of meshes to reinforce soft tissue defects and provide an additional support to prolapsed organs and viscera is a common approach to treat POP and SUI [4].

Vaginal meshes are commonly made of poly(propylene) (PP) or polyester, materials that are already used for hernia repair [5,6]. These materials are safe for hernia repair, but their safety was not properly tested for pelvic floor applications [5]. However, they were approved by the US FDA [5,6]. Since approval, multiple cases of complications associated to vaginal meshes have been reported [7]. The main problem for these meshes is the different structure and motility of the pelvic floor when compare with the abdominal wall. In addition to this, important movements and morphological changes occur during a woman’s life, and as a result, the material used to repair the pelvic floor must be not only biocompatible, but also able to tolerate the stress and tension associated with such a dynamic environment and at the same time flexible and elastic [5].

PP is the main polymer used for the production of synthetic meshes for POP surgery due to its chemical stability and non-biodegradable property [8]. However, complications such as adhesion to the viscera and high inflammatory response found in the repair of the pelvic floor [8] have led researchers to study alternative solutions. Biodegradable/bioresorbable polymers, such as poly(caprolactone) or poly(lactic acid) (PLA), have been used for mesh implant application with mixed results [9,10,11]. In some cases, this type of implants can display mechanical failures due to their degradation. For example, PLA_94_ can present mechanical problems after 8 months [11]. Accordingly, non-biodegradable polymers seem to be a safer approach. Recently, it was reported that poly(urethane)-based meshes were safer and more suitable than PP for vaginal meshes production [12,13]. Accordingly, polyurethane-based polymers seem to be the ideal candidate for this application.

In addition to safer materials, new manufacturing methods can provide benefit to the resulting medical devices. A potential technology to produce the aforementioned meshes is 3D printing. This technology allows clinicians to prepare devices adapted to patient’s anatomy and requirements [14,15,16,17]. Furthermore, a wide range of materials can be used for 3D printing applications. These materials include PLA, PP or nylon. PLA has been extensively used for biomedical applications and for 3D printing applications [18,19,20]. PLA is one of the most widely used materials for 3D printing (specifically for fused deposition modelling) [20,21]. However, as described before, due to its biodegradable nature, it is not the ideal candidate for vaginal mesh preparation. Interestingly, poly(urethane), a promising material for pelvic floor surgery, has been used before for 3D printing applications [14].

The flexibility of 3D printing also allows to combine polymeric materials with drugs to prepare drug eluting devices [14,19,22]. This is extremely useful for implantable devices that have a relatively high risk of infection [14,19,23]. If the device is loaded with antibiotics this will prevent bacterial colonisation of its surface preventing infections [14,19]. There are a wide variety of techniques within 3D printing technology [16]. In the present work, fused deposition modelling (FDM) was used. This technique relies on the extrusion of a polymeric filament trough a hot nozzle to prepare objects. To combine the polymers with drugs within the filament hot melt extrusion (HME) is needed. For this purpose, a drug substance and the selected polymer are melted inside a rotating screw to mix them and subsequently extrude them to form a filament [15]. This filament will be subsequently used for FDM applications.

The aim of this work is to develop a new generation of vaginal mesh implants. For this purpose, meshes will be prepared using thermoplastic poly(urethane) (TPU) and they will be loaded with an antibacterial agent, levofloxacin (LFX) (a drug commonly used to treat urinary infections), using fused deposition modelling (FDM). This technique is the most common type of 3D printing. Three different filaments of TPU containing 0.25%, 0.5%, and 1% of LFX were prepared through single hot-melt extruder in order to be used for the 3D Printing FDM process. Mechanical strength, drug release and antimicrobial properties were evaluated to confirm the efficiency of the meshes.

## 2. Materials and Methods

### 2.1. Materials

Elastollan^®^ thermoplastic polyurethane (TPU) 80A pellets were used for this study and kindly provided by DistruPol Ltd. (A Univar Company, Co., Dublin, Ireland). Castor oil was purchased from Ransom, LFX ((S)-9-fluoro-2,3-dihydro-3-methyl-10-(4-methylpiperazin-1-yl)-7-oxo-7H-pyrido[1,2,3-de]-1,4-benzoxazine-6-carboxylic acid) >98% was obtained by Sigma–Aldrich, and the phosphate buffered saline (PBS) tablets pH 7.4 from Merck. The PP filament (2.85 mm diameter) was purchased from Verbatim (Tokyo, Japan).

### 2.2. Preparation of Thermoplastic Poly(urethane) (TPU) Filaments Containing Levofloxacin (LFX)

In order to 3D print meshes, filaments were prepared using the Hot-Melt Extrusion (HME) technique by combining the TPU with LFX. An oil method was used to ensure a homogeneous distribution of the drug on the pellet’s surface. TPU pellets (30 g) were placed in 50 mL Falcon tubes and castor oil (30 µL) was added and vortexed for a few min in order for the pellets to be covered homogeneously by the oil. The pellets were transferred to a new 50 mL Falcon tubes to avoid drug wastage that could remain attached due to excess oil on the wall of the previous tubes, as previously reported [14]. Then, LFX was added in ratio of 0.25% *w*/*w* and the tube was vortexed in order to coat the pellets. Finally, the coated pellets were introduced in the filament extruder (3Devo, Utretch, The Netherlands) using an extrusion speed range of 3–5 rpm and a filament diameter of 2.85 mm. The temperature was regulated directly during the extrusion over four heaters between 170 °C and 200 °C. The same procedure was performed for preparing filaments containing 0.5% and 1% of LFX. The filament formed using only TPU, which used for the preparation of blank meshes, was manufactured introducing directly the pellets into the extruder. Formulations with their compositions to manufacture the filaments are presented in the Table 1.

### 2.3. Preparation of 3D Printed Meshes Containing LFX

Meshes were printed with the drug-loaded and unloaded filaments that previously prepared with the extruder, using an Ultimaker 3 (Ultimaker B.V., Geldermalsen, The Netherlands) fused filament fabrication (FFF) system, furnished of two extruders with a 0.4 mm nozzle, and Cura® software 3.0 (Ultimaker B.V., Geldermalsen, The Netherlands). Different models were designed through a CAD-based software. For the TPU meshes, the layer height was set at 0.1 mm with the in-fill setting on the software at 100%. The printing temperature was set at 190 °C and the printing speed was 12 mm/s. However, for the PP meshes, the printing temperature was set between 195 °C and 208 °C and the printing speed was 25 mm/s. These PP meshes were manufactured using the filament obtained from Verbatim (Tokyo, Japan).

### 2.4. Characterization of 3D Printed Meshes

#### 2.4.1. Mechanical Properties

Meshes with 50 mm × 10 mm size were printed, and the fracture force was studied with TA.XTplus texture analyser (Stable Micro Systems, Surrey, UK). Each sample was vertically fixed with two clamps, with a distance between them of 40 mm, and stretched at a rate of 5 mm/s up to 200 mm. The experiment was repeated 4 times for each sample. The force/displacement curves were recorded, and different parameters were obtained. The elastic limit of the resulting meshes were obtained using the 0.2% offset method [24]. Additionally, the tensile stiffness was calculated from the force/displacement curves as the slope of the initial linear region [25].

#### 2.4.2. Fourier Transform Infrared (FT-IR) Spectroscopy

The Fourier Transform Infrared (FT-IR) spectra of 1 cm × 1 cm meshes were recorded through a Spectrum Two™ instrument (Perkin Elmer, Waltham, MA, USA). The spectra were recorded between 4000 cm^−1^ and 600 cm^−1^ applying a resolution of 4 cm^−1^; total of 32 scans were collected.

#### 2.4.3. Thermogravimetric Analysis (TGA)

As the elastomer was subjected to high temperatures during the 3D-printing process, the thermal degradation behaviour of the polymer was examined. Thermogravimetric analysis (TGA) was performed to measure the weight loss of the TPU meshes containing LFX. For this purpose, a small fragment of these meshes (3 mg and 10 mg) was used. TGA was performed using a Q500 Thermogravimetric analysis (TA instruments, Bellingham, WA, USA). Scans were run at room temperature to 550 °C, at a speed rate of 10 °C/min under a nitrogen flow rate of 50 mL/min.

#### 2.4.4. Scanning Electron Microscopy (SEM)

Scanning electron microscopy was used in order to investigate the surface morphologies of the 1 cm × 1 cm 3D-printed meshes containing 0.25%, 0.5% and 1% of LFX compared with the blank mesh, using samples before and after a 14-day release study. The meshes were examined using a Hitachi TM3030 SEM (Tokyo, Japan), and images were taken with a magnification of 50×, 60×, 80×, 300× and 500×. Additionally, a Leica EZ4 D digital microscope (Leica, Wetzlar, Germany) was used to examine the presence or not of drug aggregates within the extruded materials.

#### 2.4.5. X-ray Microcomputed Tomography (μCT)

X-ray Microcomputed Tomography imaging was performed on 3D printed meshes using the same approach previously reported [14]. Briefly, all the samples were analysed by using a Bruker Skyscan 1275 (Bruker μCT, Kontich, Belgium), with a Hamamatsu L11871 source (40 kV, 250 µA). The meshes were mounted vertically on dental wax and positioned at 57.5 mm from the source, where camera to source distance was 286 mm. No filter was applied for an exposure time of 49 ms. The images generated were 1536 × 1944 pixels with a resolution of 17 µm per pixel. A total of 1056 images were taken in 0.2° steps around one hemisphere of the sample, with an average of 3 frames taken at each rotation step. Attenuation thresholding was conducted manually, in order to eliminate speckle around the samples. The same thresholding was applied within Bruker’s CTAn software v.1.18, where the samples were further processed.

### 2.5. In Vitro Drug Release Studies

The release profile for the LFX was defined conducting release studies that allowed calculating the amount of drug eluted from the LFX-loaded meshes. Each sample was placed in Eppendorf’s with 2 mL of PBS. Subsequently the Eppendorf’s were located in a shaking incubator at 37 °C at 40 rpm. After 1, 2, 4, 24, 48, 72, 96 and 120 h the sample was removed from the tube, dried and relocated in a new Eppendorf containing 2 mL of fresh PBS. Further studies performed also in new samples for 7 and 14 days. The concentration of LFX was calculated after measuring the UV absorbance of the solution taken from the Eppendorf’s with a UV–visible plate reader (PowerWave XS Microplate Spectrophotometer, Bio-Tek, Winooski, VT, USA) at a wavelength of 292 nm as previously reported [26]. For each concentration (control, 0.25%, 0.5% and 1%), 1 cm × 1 cm meshes were used in series of 4.

### 2.6. In Vitro Microbiological Analysis

Printed meshes (1 cm × 1 cm × 0.1 cm) were tested for inhibitory effect on bacterial cultures of *Staphylococcus aureus* NCTC 10788 (Gram-positive) and *Escherichia coli* NSM59 (Gram-negative). *E. coli* and *S. aureus* are examples of bacteria that can cause a variety of community-and hospital-acquired infections. This in vitro microbiological analysis was performed according to a previous published work, with some modifications [14]. Briefly, bacteria were grown overnight at 37 °C in Mueller–Hinton (MH) broth. For each bacterium, 50 µL of the overnight culture were added to 5 mL of MH soft agar. This mixture was vortexed and then poured on top of the MH agar plate. Finally, meshes were placed in the centre of the plate and incubated for 24 h at 37 °C. The inhibition zone caused for both bacterial strains was then measured in mm. Moreover, inoculated plates for each bacterial strain were also incubated as a positive control. The results were expressed as mean ± standard deviation of 5 replicates.

### 2.7. Statistical Analysis

Quantitative data were expressed as a mean ± standard deviation, *n* ≥ 3. The statistical analysis was performed using a one-way analysis of variance (ANOVA), *p* < 0.05 was considered to be statistically significant.

## 3. Results

### 3.1. Preparation and Characterisation of TPU Filaments and Meshes Containing LFX

**Figure 1:**
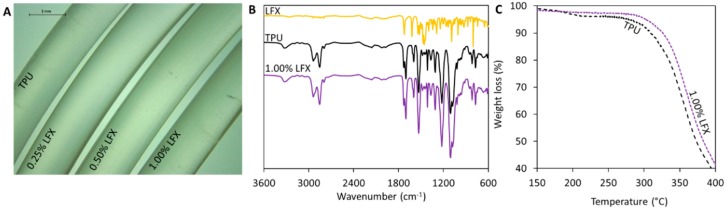
Microscopy image of the thermoplastic polyurethane (TPU) and levofloxacin (LFX)-loaded TPU filaments (**A**). FTIR spectra of LFX, TPU and TPU containing 1% of LFX (**B**). TGA of TPU and TPU containing 1% LFX (**C**).

The extrusion of the TPU pellets containing the different LFX concentrations were used to produce smooth and flexible filaments of 2.85 mm in diameter (Figure 1A). The resulting materials contained different amounts of LFX ranging from 0.25% to 1% (*w*/*w*). All the filaments prepared using hot-melt extrusion showed the same translucent colour. No visible aggregates of drug were seen within the extruded materials. Considering that LFX is a white solid, this suggests that the antibiotic was mixed with the molten TPU within the extrusion process. Moreover, the results suggest that TPU and LFX can be mixed properly using a single screw extruder following the pellet coating method. Otherwise, more complicated equipment—such as a twin-screw extruder—will be required to mix the drug and the polymer properly.

FT-IR and TGA were used to try to establish if there was any interaction between TPU and LFX. The FT-IR spectra of the materials containing LFX showed the same peaks as the blank TPU (Figure 1B). The drug loadings selected for the present work were too low to be able to produce any changes in FT-IR spectra. However, TGA measurements (Figure 1C) show that when LFX was combined with TPU using hot melt extrusion, the resulting material presented different thermal degradation behaviour. Filaments containing LFX started to degrade at higher temperatures than the blank TPU filaments. In order to compare both materials, the onset temperatures (T_onset_) were measured. T_onset_ denotes the temperature at which the weight loss begins (5% weight loss). The onset temperature for TPU was 280 °C, while the recorded onset temperature for TPU containing 1% of LFX was 303 °C. As mentioned before this temperature differences can be attributed to interactions between the TPU and the LFX.

**Figure 2:**
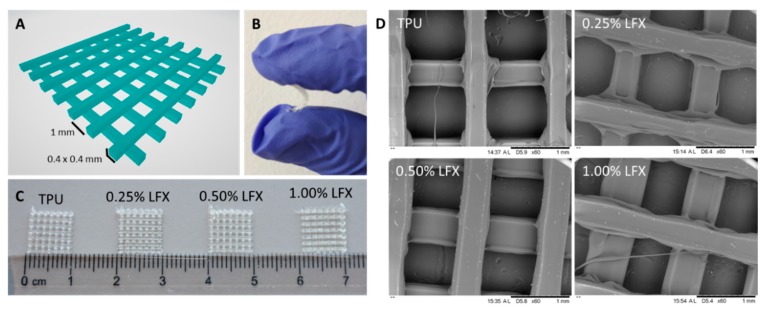
CAD 3D image of the two layer meshes with its dimensions (**A**). Representative image showing the flexibility of a TPU-based mesh (**B**). Image of TPU and TPU loaded with LFX 3D printed meshes (**C**). SEM images of TPU and LFX loaded TPU 3D printed meshes (**D**).

The TPU filaments previously described were used to prepare different types of surgical meshes. These designs were prepared using Computer Aided Software and subsequently prepared using fused deposition modelling. Figure 2A shows the designs used to prepare the meshes with their dimensions. Moreover, Figure 2C shows some 1 × 1 cm mesh prototypes produced using the filaments described in Section 3.1. As expected, all these prototypes presented the same appearance as LFX was completely mixed with the TPU. These resulting meshes are flexible as can be seen in Figure 2B. These results can be corroborated by using SEM (Figure 2D). The microscopy images showed that all the resulting meshes showed the same structure and no signs of drug aggregation within the surface of the devices.

**Figure 3:**
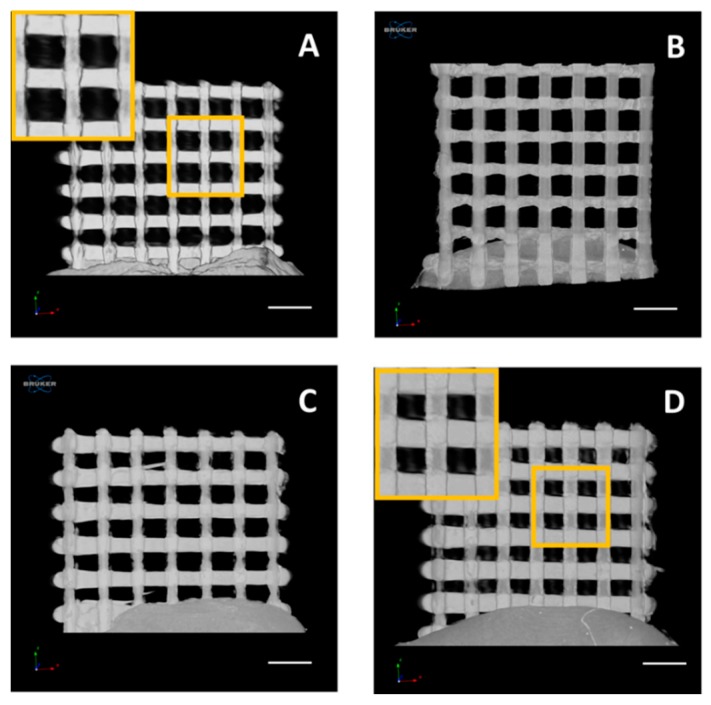
µCT reconstructions in the xz plane of pure TPU80 mesh (**A**) and TPU80 mesh loaded with 0.25% (**B**), 0.5% (**C**) and 1% (**D**) of LFX (scale bar = 2 mm).

The 3D printed samples were analysed by using a Bruker Skyscan 1172 system (Figure 3), in order to investigate samples’ topology as well as drug distribution within their architecture. As it could be seen in Figure 3B–D, the incorporation of LFX did not affect the 3D printed mesh morphology, which resulted very similar for all the analysed samples and comparable to the one of pure TPU80 (Figure 3A).

In addition, as shown in the representative reconstruction images, the meshes exhibited the same topology. Particularly, even at the highest concentration of LFX (Figure 3D), no traces of particles were detected within the printed meshes, thus indicating a uniform distribution of the drug, regardless the concentration tested. Moreover, according to this outcome it was further demonstrated the effectiveness of the manufacturing process from drug incorporation to 3D printed sample fabrication.

### 3.2. Mechanical Characterisation of LFX 3D Printed Meshes

**Figure 4:**
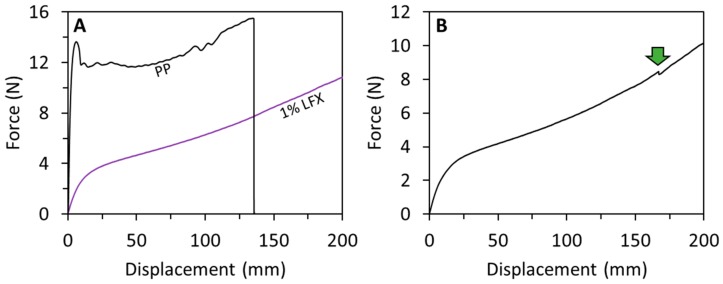
Force/displacement graphs obtained for TPU meshes containing 1% LFX and PP meshes (**A**). Force/displacement graph showing a small fracture for a TPU-based mesh (**B**). The arrow indicates the fracture point.

The mechanical properties of two-layered mesh implants prepared using fused deposition modelling were measured. Figure 4 shows representative force/displacement graphs for the prepared meshes. All the TPU-based meshes showed similar profiles. The first region of the graph showed elastic behaviour (initial linear section of the graph), and then when higher forces were applied the meshes showed plastic deformation (see Figure 4A,B). It is important to note that though they did not fully break under the testing conditions (200 mm of elongation), in some cases they show some minor fractures during the last stages of the test (Figure 4B). However, this does not happen consistently in all the meshes. This was observed only in two cases. It is important to note that these partial fractures happened after the mesh elongated more than three times its original size. On the other hand, meshes made of PP were prepared to compare the obtained results with the material typically used for mesh implant manufacturing. PP showed a different mechanical behaviour than TPU-based meshes. PP meshes failed during the test as they showed a clear and reproducible fracture point (Figure 4A).

The elastic limit and the tensile stiffness were evaluated from the force/displacement curves. The elastic limit was measured from the force/displacement curves using the 0.2% offset method. This value represents the force required to produce a 0.2% of plastic deformation of the meshes. All TPU-based meshes showed elastic limits around 1 N (Table 2). Moreover, a statistical analysis showed that there were no significant differences between all these values (*p* > 0.05). These results suggest that LFX loadings of up to 1% (*w*/*w*) did not alter the mechanical properties of TPU. This is important for future applications, as TPU was selected due to its elasticity as opposed to conventional PP meshes. Polypropylene meshes showed significantly higher elastic limit than the TPU-based meshes (*p* < 0.05). This is consistent with the nature of the material that is not an elastic material as opposed to TPU. Finally, the tensile stiffness of the mesh implants was evaluated. Again, the results showed that all TPU-based meshes showed equivalent values of tensile stiffness ca. 0.4 N/mm (*p* > 0.05). Moreover, PP meshes showed significantly higher values of tensile stiffness (*p* < 0.05). These values showed that PP required higher forces to elongate within the elastic region of the material. Accordingly, PP is a tougher material with lower elasticity. Again, TPU seems a more suitable approach for mesh implant manufacture due to its elasticity.

### 3.3. LFX Release from 3D Printed Meshes

**Figure 5:**
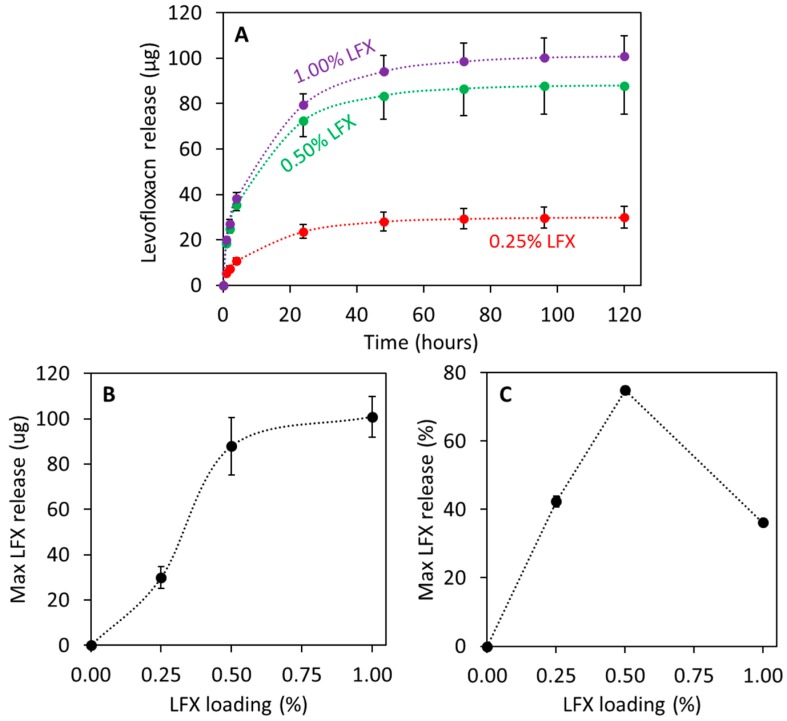
LFX release as a function of time for different LFX loaded 3D printed meshes (**A**). Maximum LFX release expressed in µg (**B**) and percentage (**C**) as a function of initial LFX drug loading.

Figure 5 shows the LFX release from 3D printed meshes. Figure 5A shows the LFX released as a function of time for the 3D printed meshes. The prepared meshes are capable of providing sustained release of LFX for at least 3 days. Additionally, it can be seen that all the release profiles showed the similar shapes. The total amount of LFX released after 5 days (Figure 5B) increased with drug loading. However, there is a significant increase in the drug loading when the LFX loading increased from 0.25% to 0.5% (*p* > 0.05). When drug loading increased from 0.5% to 1% a small increment in drug release was observed. However, statistical analysis revealed that this different is not statistically significant (*p* < 0.05). Accordingly, it can be hypothesised that LFX could be interacting with TPU within the meshes, preventing a higher drug release. This is consistent with the results described in Section 3.1. These results are more obvious when the release was expressed as percentage of the initial drug loading (Figure 5C). This graph showed some interesting results. The percentage of drug release increase with drug loading up to a maximum. This maximum was obtained for meshes containing 0.5% of LFX. Subsequently, the percentage of drug release decreases when drug loading was increased up to 1% (*p* < 0.05). This showed that LFX/TPU interactions are taking place and reducing drug release.

### 3.4. Antimicrobial Properties of LFX Loaded 3D Printed Meshes

**Figure 6:**
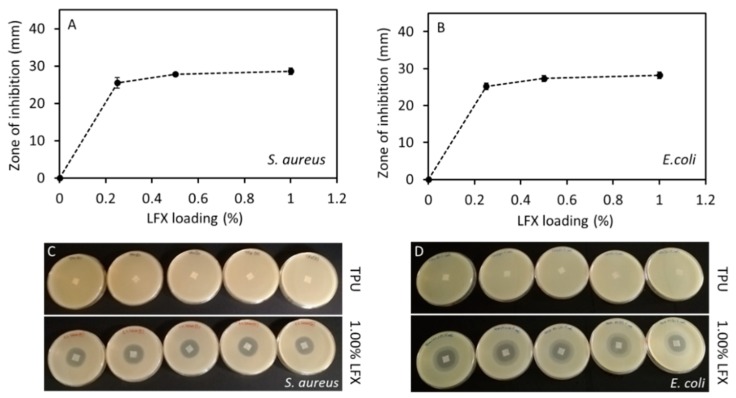
Correlation between the diameter of the zone of inhibition of *S. aureus* (**A**) and *E. coli* (**B**) and the concentration of LFX. Agar plates showing the zone of inhibition of meshes without LFX (TPU) and containing 1% of LFX for both bacterial strains, *S. aureus* (**C**) and *E. coli* (**D**).

Printed meshes (1 cm × 1 cm × 0.1 cm) containing different LFX concentrations were tested for antimicrobial effect on a bacterial culture of *S. aureus* and *E. coli* in order to evaluate good examples of bacteria that are involved in a variety of community-and hospital-acquired infections. The results of the zone of inhibition are presented in the Figure 6. In this case, the zone of inhibition indicates that both used bacteria either at the surface of the meshes or even for an area extending outwards from the mesh’s surface is inhibited. All the meshes containing LFX showed a clear zone of inhibition in both *S. aureus* and *E. coli* plates. As expected, the results showed no zone of inhibition in plates containing the control meshes without LFX.

The zones of inhibition in both *S. aureus* and *E. coli* plates were increased by increasing the amount of LFX. The diameter of the zone of inhibition in the *S. aureus* plates with TPU meshes containing LFX ranged from 25.5 ± 1.4 mm to 28.6 ± 0.8 mm, and from 25.2 ± 0.9 to 28.2 ± 0.8 in the *E. coli* plates. Statistical analysis showed that there were significant differences between the zones of inhibition caused by meshes containing 0.25% and 0.5% or 1% LFX (*p* < 0.05). This behaviour was observed for both cultures, *S. aureus* and *E. coli*. However, there were no significant differences in the zone of inhibition caused by meshes containing 0.5% and 1% LFX (*p* > 0.05). Once again, this trend was observed for both bacterial strains. These results are is in line with the obtained drug release profile for the meshes containing LFX (Figure 5A). In addition, when the zones of inhibition of *E. coli* and *S. aureus* were compared for the same concentration of LFX (0.25%, 0.5% and 1%), no significant differences were observed for any LFX concentration (*p* > 0.05). Therefore, it can be inferred that LFX had the same impact on both bacterial strains, which are the most frequent causes of many common bacterial infections.

## 4. Discussion

Historically, PP has been the choice material for pelvic floor repair since 1995 [13]. However, it has been shown that this material is not the ideal candidate for these applications due to the mechanical mismatch between the elastic paravaginal tissue and the strong and rigid PP [27]. Accordingly, the mechanical properties of PP mesh have generated multiple problems after mesh implantation. According to the US FDA, the use of PP mesh for pelvic floor repair can lead to serious complications associated with tissue erosion [28,29]. The ideal material for the production of pelvic floor repair mesh implants should possess elasticity and strength [12].

The present work describes the use of fused deposition modelling for the production of mesh implants for potential pelvic organ reconstructive surgery. TPU was selected as the ideal candidate for this purpose due to its elasticity and previously demonstrated biocompatibility [12,13,18]. This material has been used before for mesh implant manufacturing, showing superior capabilities than PP implants [12,13]. Additionally, TPU was combined with an antibiotic drug to prevent infection of this implantable material after surgery. Mesh-related infections are not common but when they occur they can compromise patients’ well-being even leading to excision of the mesh implant or sepsis [30].

LFX was the antibiotic chosen for this application. In a previous work it was loaded in meshes prepared using electrospinning for hernia repair [26]. This antibiotic was combined with TPU using hot-melt extrusion to prepare filaments for further FDM applications. The materials displayed the homogeneous distribution of the drug. This was achieved using a single screw extruder coating the TPU pellets with LFX. This method has been previously used with successful results [14,19,31,32]. This is a quick way to obtain good mixtures between the drug and the polymer using a single screw extruder that is more accessible than a complicated and expensive twin-screw extruder. Figure 1A shows that the drug was properly dispersed within the material. FTIR results did not show any noticeable peak shift (Figure 1B). As mentioned before this can be due to the low drug loading. Similar behaviour was reported before for the combination of TPU and tetracycline or poly(urethane) and ciprofloxacin, a drug similar to LFX [14,33]. On the other hand, TGA results (Figure 1C) shows that there was interaction between LFX and TPU. Similar behaviour was reported when TPU was combined with tetracycline, ciprofloxacin or Schiff base additives [14,33,34]. It has been proposed that the C=O groups present in the TPU urethane groups can stablish non-covalent interactions with the drug.

The interaction of LFX with TPU can explain the behaviour obtained in the drug release profiles. In these experiments, meshes containing 1% of LFX showed a lower percentage of LFX released from the meshes than meshes containing 0.5% of LFX. The interactions between the polymer and the drug prevents a higher drug release. This has been observed previously for other drugs such as dipyridamole loaded into polyurethane [35]. Similarly, lower drug loadings (0.25% LFX) showed low release too. TPU is a non-degradable/hydrophobic polymer and, accordingly, the drug cargo located inside the material will not be released. Finally, the TPU meshes described in the present work are capable of providing releases of LFX for at least 3 days. A previously published work describing the use of electrospinning to prepare poly(caprolactone) surgical meshes loaded with LFX (0.5%) showed that this system was capable of providing drug release over l day. However, the nature of the mesh forming polymer was completely different.

This work was not only focused on the development of safer materials for mesh implant manufacturing but the use of techniques that allow clinicians to customize the mesh to patient’s needs in a simple way. Therefore, FDM seems like an ideal technique for this purpose. TPU based meshes were successfully prepared using FDM (Figure 2). As expected, all the meshes had the same appearance and now noticeable drug aggregation was observed (Figure 2). Computed tomography was used to confirm drug distribution within the mesh matrix. Again, the results suggested that the drug was uniformly distributed within the mesh. In a previous study, computed tomography suggested that the combination of similar TPU with tetracycline showed some drug accumulation in certain parts of the material [14]. In this case, tetracycline was distributed all over the material, but some accumulation was observed using computed tomography.

The observed mechanical properties of the resulting meshes proved the initial approach: the resulting materials showed elastic behaviour unlike PP. The TPU-based meshes showed stiffness values ca. 0.4 N/mm while commercial PP meshes showed values ranging between 2 and 6 N/mm [25]. The design of the commercial meshes is different than the one proposed in the present paper but the testing conditions for these commercial meshes were similar. Some comparisons can be made. In order to compare the effect of the material in the mechanical properties, PP meshes were prepared using the same design used for the TPU based materials. Obviously, this PP is not exactly the same as the one used in conventional meshes, but it is a good example to compare the behaviour of both materials. The stiffness results obtained for PP (ca. 6 N/mm) were higher than those obtained for TPU meshes and the Force/displacement profile was completely different. Moreover, the stiffness values obtained for PP meshes were slightly higher than the previously reported results for commercial PP meshes (up to 5.3715 N/mm) [25]. However, the PP meshes tested in this work showed a different design than the commercial meshes. The mechanical characteristics of the material are important as it has been reported that materials with higher flexibility seem to adhere and conform to the tissues better than more rigid/stiffener meshes [36]. The design and size of the meshes can be hanged easily due to the versatility of FDM.

The 3D-printed meshes had a bacteriostatic activity on both *S. aureus* and *E. coli* cultures (Figure 6). This fact supports the premise that the extrusion and 3D printing processes did not affect the bacteriostatic activity of LFX. The risk of toxicity of these coated medical devices could be an important issue. Therefore, the possibility to print these medical devices using a small amount of the desired drug, and still have bacteriostatic activity, clearly minimizes the risk of toxicity in the patients. For instance, medical devices such as thermoplastic polyurethane (TPU) catheters were 3D-printed using up to 1% of tetracycline [14], thereby minimizing the risk of infection. Furthermore, Weisman et al. [31], in a different study, reported the possibility to print poly(lactic acid) (PLA) catheters using up to 2.5% of gentamicin. Additionally, it is also possible to print medical devices using higher percentages of drugs. Thus, for example, Genina et al. [37] 3D-printed drug-loaded intrauterine devices using different grades of ethylene vinyl acetate containing 5% and 15% of indomethacin.

PLA pellets coated with 1 wt % gentamicin were used to fabricate mesh prototypes for hernia repair [38]. In this study, they obtained a zone of inhibition of 1.1 ± 0.1 cm^2^ for *E. coli* and 1.2 ± 0.1 cm^2^ for *S aureus*. In a different work, polyvinyl alcohol (PVA) 3D meshes loaded with iodine were manufactured and these also showed a zone of inhibition against *E. coli* and *S. aureus* [39]. These results were far below to those found in our work. The diameter of the zone of inhibition in the *S. aureus* plates with TPU meshes of 0.25% LFX was 25.5 ± 1.4 mm and 28.6 ± 0.8 mm for meshes containing 1% LFX. As mentioned above, there were no significance differences between these results and the ones obtained in the *E coli* plates (*p* > 0.05). Therefore, it can be inferred that even the lower concentration (0.25%) of LFX had a significant zone of inhibition on both bacterial stains, which further minimises the risk of toxicity.

The use of medical devices such as transvaginal meshes, catheters or ventilators could be associated with the development of “nosocomial” or “health-care associated infections” (HCAIs) [40,41]. Although bacteria, viruses or fungal parasites can cause these infections, bacteria are the most common pathogens responsible for HCAI. Among these, bacterial species as *S. aureus* and *E. coli* have a major impact [42]. *S. aureus* is one of the most important pathogens responsible for nosocomial infections [43]. Moreover, *E. coli* is an emerging nosocomial pathogen, which is the leading cause of urinary tract infections (UTI) while, *S. aureus* is rarely found in these infections [43,44]. These infections may result in prolonged stays in the different health-care facilities, such as hospitals while increasing health-care costs [45]. Hence, the use of these 3D-printed meshes could decrease the rate of bacterial infections caused by the implant.

The majority of the FDM applications describing the combination of polymers with drugs are focused on the development of oral solid dosage forms [46,47]. We believe that this technology has the potential to be used for the manufacturing of medicated devices that can be produced on demand for a patient before a specific treatment/surgery. Previously we reported the use of FDM for dialysis catheter manufacturing [14,19] or antioxidant wound dressings. Some preliminary work has been done about the use of 3D printing for mesh implant manufacture. However, these works were not realistic as they propose the use of materials such as PLA or PCL that are biodegradable and do not present appropriate mechanical properties for this task [38,48,49]. Some of these works incorporated some antibiotics to the material. However, these works were not realistic due to material selection, but these studies worked as a proof of concept showing the potential of 3D printing for this purpose. Additionally, some recent work described the potential of using FDM as a tool for mesh implant manufacturing using PP [50]. The limitations of this material have been described previously. Moreover, these authors incorporated ciprofloxacin into the meshes by dip coating the implants. This is not ideal, as the manufacturing involves a two-step process. In the present work, the mesh is produced directly containing the drug within the device. Further research needs to be conducted about the in vivo biocompatibility of the meshes and shape optimization to adapt the mechanical properties of the mesh to patient’s needs. The present work is a proof of concept that shows the potential of FDM technology to prepare elastic anti-infective materials. Finally, there are still regulatory aspects that should be addressed before 3D printing can be approved as a manufacturing technology for surgical devices. The US FDA has published some guidelines to manufactures about the appropriate use of this technology [51].

## Figures and Tables

**Table 1:** Composition of TPU filaments containing LFX.

Formulations	TPU (g)	Castor Oil (μL)	LFX (g)
TPU	30	-	-
0.25% LFX	30	30	0.075
0.50% LFX	30	30	0.15
1.00% LFX	30	30	0.3

**Table 2:** Mechanical properties obtained for the 3D printed meshes formed by two layers.

	LFX Content (%)	Elastic Limit (N)	Tensile Stiffness (N/mm)	Fracture Force (N)	Elongation at Break (mm)
TPU	0.00	1.2 ± 0.4	0.44 ± 0.12	-	-
LFX 0.25%	0.25	1.0 ± 0.2	0.32 ± 0.06	-	-
LFX 0.50%	0.50	1.1 ± 0.1	0.37 ± 0.04	-	-
LFX 1.00%	1.00	1.3 ± 0.2	0.45 ± 0.08	-	-
PP	0.00	6.5 ± 0.2	6.05 ± 0.83	15. 42 ± 0.66	129 ± 7
